# ProGeRF: Proteome and Genome Repeat Finder Utilizing a Fast Parallel Hash Function

**DOI:** 10.1155/2015/394157

**Published:** 2015-02-25

**Authors:** Robson da Silva Lopes, Walas Jhony Lopes Moraes, Thiago de Souza Rodrigues, Daniella Castanheira Bartholomeu

**Affiliations:** ^1^Department of Computer Science, Federal University of Mato Grosso, 78600-000 Barra do Garcas, MT, Brazil; ^2^Federal Center of Technological Education of Minas Gerais, Belo Horizonte, MG, Brazil; ^3^Department of Parasitology, Federal University of Minas Gerais, 31270-829 Belo Horizonte, MG, Brazil

## Abstract

Repetitive element sequences are adjacent, repeating patterns, also called motifs, and can be of different lengths; repetitions can involve their exact or approximate copies. They have been widely used as molecular markers in population biology. Given the sizes of sequenced genomes, various bioinformatics tools have been developed for the extraction of repetitive elements from DNA sequences. However, currently available tools do not provide options for identifying repetitive elements in the genome or proteome, displaying a user-friendly web interface, and performing-exhaustive searches. ProGeRF is a web site for extracting repetitive regions from genome and proteome sequences. It was designed to be efficient, fast, and accurate and primarily user-friendly web tool allowing many ways to view and analyse the results. ProGeRF (Proteome and Genome Repeat Finder) is freely available as a stand-alone program, from which the users can download the source code, and as a web tool. It was developed using the hash table approach to extract perfect and imperfect repetitive regions in a (multi)FASTA file, while allowing a linear time complexity.

## 1. Introduction

Repetitive elements are found in large quantities in eukaryotic genome, both in coding and noncoding region, and also in intergenic regions of prokaryotes [1]. In humans repetitive elements represent approximately 7% of the genome [2]; in parasites protists the proportion of repetitions varies from 11% to 65% of the DNA [3] while in protozoa such as* Theileria parva*,* Plasmodium berghei*,* T. cruzi,* and* Toxoplasma gondii*, this value varies between 4% and 30% of repeating sequences in genomes [4, PMID: 16020725].

Repetitive sequences can be categorized into two groups: interspersed repeats and tandem DNA repeat. Interspersed repeats are mainly active or inactive copies of transposable elements dispersed throughout the genome and are divided into DNA transposons and retrotransposons [5], while the tandem repeats are ribosomal DNA sequences and satellite DNA [4, 6].

Normally, tandem repeats are classified according to the repetitive motifs length in microsatellites, minisatellites, and macrosatellites. Microsatellites (also known as short tandem repeats (STRs) or simple sequence repeats (SSRs)) are small stretches of DNA sequences (usually <200 bp), with motif lengths between 1 and 6 bp. Minisatellites are large repetitive sequences, with motif lengths of 5 to 25 bp, and the macrosatellites are large regions of repeats with lengths larger than 25 bp [4, 7, 8].

Microsatellites can be classified as perfect, imperfect, and compound. Perfect repetitive elements are formed from identical repetitive units. Imperfect repetitive elements are units with small mutations and may have been caused by insertions, deletions, or replacements. Repetitive compounds elements are composed of sequences in which two or more repetitions (perfect or imperfect) are arranged successively with or without nucleotide bases between them [8].

Repetitive elements, mainly microsatellites, have been widely used as molecular markers in phylogenetic studies, analyses of genetic populations, construction of genetic maps, paternity testing, and forensic medicine [7, 9]. The main explanation given for the emergence of variation in the amount of repetitions is a sliding model (slippage) of DNA polymerase during DNA replication [10].

Given the importance of identifying repeating regions and the possibility of identifying them in silico, many tools for identifying repeating regions have been developed. Work carried out by Lim et al. [2], Mudunuri et al. [8], and Leclercq et al. [1] reviewed and tested the main tools for identifying repeats. The following are the most commonly used tools for extracting repeat regions of a genome: TRF [11], TROLL [12], Misa [13], Mreps [14], SciRoKo [15], Sputnik [16], SSR Locator [17], IMEX [18], and GMATo [19].

However, it should be taken into consideration that all of the above software tools are unable to obtain all of the possible sequences because they (a) locate only perfect repetitions (GMATo, TROLL, and Misa); (b) make use of probabilistic or statistical patterns heuristics that do not meet all possible repetitions (TRF and Mreps); and (c) are unable to execute on large FASTA files (SciRoKo and Mreps). Finally, none of these tools can be executed in both DNA and protein datasets.

Thus, this paper presents a fast and efficient algorithm inspired by the concepts of “Sequence Search and Alignment by Hashing Algorithm,” SSAHA [20], that stores information about the locations of DNA words into a hash table and based on circular doubly linked lists for a fast and exhaustive identification of repetitive elements, both perfect and imperfect, in large DNA or protein FASTA files.

## 2. Methods

### 2.1. Definitions

Some definitions are presented below to facilitate understanding.

*Sliding Window Method*. To identify a given full-length DNA or protein sequence, the sliding window approach is employed to obtain sequences with variable length, where *Q* represents the sequence obtained for a sliding window and is called a DNA or amino acid word and |*Q*| is word length.

*Hash Table*. This consists of an array where the data to be searched is stored and is accessed via a special index called a *key*. In our case, we store information about each motif. Hash table is allocated dynamically for each motif and there are *r* ^|*Q*|^ positions, where *r* is the radix (four for DNA and twenty for amino acids), and |*Q*| is the length of the word (which in our case is the sliding window length). With this, the hash table can have a position for each combination of nucleotides or amino acids of size |*Q*|.

*Hash Function*. A hash function that maps DNA or amino acids to digits is based on the [21] conversion, where a hash function *m* is defined as a function that maps each DNA base or amino acid into digits, which in turn corresponds to a position (index) in the hash table. For DNA, each nucleotide is mapped as *m*(A) = 0, *m*(C) = 1, *m*(G) = 2, and *m*(T) = 3 and for amino acid residue it is mapped as *m*(G) = 0, *m*(P) = 1, *m*(A) = 2, *m*(V) = 3, *m*(L) = 4, *m*(I) = 5, *m*(M) = 6, *m*(C) = 7, *m*(F) = 8, *m*(Y) = 9, *m*(W) = 10, *m*(H) = 11, *m*(K) = 12, *m*(R) = 13, *m*(Q) = 14, *m*(N) = 15, *m*(E) = 16, *m*(D) = 17, *m*(S) = 18, and *m*(T) = 19. The DNA or amino acid word (sliding window) is converted into a number applying the general positional number system conversion function *h*() to *Q*_*p*_ = {*q*_0_ *q*_1_ *q*_2_ ⋯ *q*_|*Q*|−1_}, where *h*() is defined by

(1) $$\begin{array}{c} h\left(Q_{p}\right)=\overset{\left|Q\right|-1}{\underset{i=0}{\sum }}m\left(q_{i}\right)r^{\left(\left|Q\right|-1\right)-i}. \end{array}$$

Here *Q* is a DNA or amino acid word, *m* is the hash function, *q* is one base of word, *p* is the DNA or amino acid word start position on the sequence, *r* is the radix (four for DNA and twenty for amino acids), and |*Q*| is the length of the word (which in our case is the sliding window length). For instance, the DNA word ACTGC is (0∗4^4^) + (1∗4^3^) + (3∗4^2^) + (2∗4^1^) + (1∗4^0^) = 121.

*Single Bucket*. It consists of a 5-tuple, in which the information of each repetitive pattern for a given motif is recorded. It is formed by 〈*sp*, *fp*, *mt*, *g*, *lt*〉, where *sp* and *fp* are the initial and final positions of the repetitive pattern, respectively, *mt* is the repetitive motif, *g* is the amount of gaps within the repetitive sequence, and *lt* is the number of repetitions of the motif *mt* inside of this substring. Each index *k* of the repetitive elements hash table contains a list of single buckets, where every single bucket represents a repetitive sequence of motifs mapped to the value *k*. A circular doubly linked list has been utilized to implement the list of single bucket lists, thus ensuring the insertion and deletion of a bucket quickly.

### 2.2. Architecture

**Figure 1:**
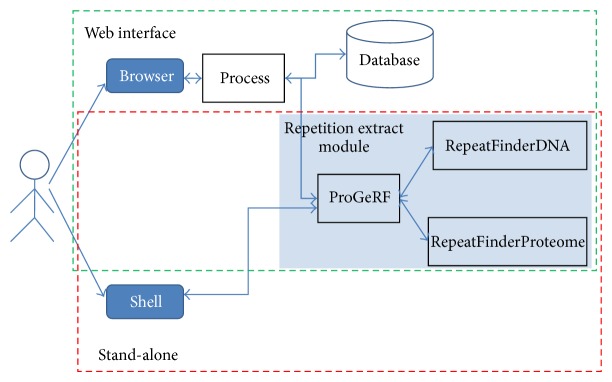
ProGeRF architecture. Structure of the tool both for the web environment and for the stand-alone mode. The dark blue rectangles with rounded corners represent interfaces with the system. The transparent rectangles with a blue background represent algorithms done in C or Perl. The process script receives data from the web environment, treats the data, saves them in a MySql database, and calls the repetition extract module.

ProGeRF is available in two execution modes, as illustrated in Figure 1: as a stand-alone program, from which the users can download the source code, compile, and run in their machine in a Linux environment and as a web tool available at the web address http://64.79.105.19/ligp/. At this address, it is also possible to download the stand-alone version.

Repeat extraction module has been used in this two execution modes. This module consists of three algorithms: one developed in Perl and two developed in C language. The perfect and imperfect repetitions are identified by algorithms in C language, called RepeatFinderDNA and RepeatFinderProteome. The first algorithm works on a FASTA file with DNA sequences and the second algorithm works on a FASTA file with amino acids sequences.

The Perl script, called ProGeRF, receives the input parameters, performs the call to the RepeatFinder algorithms, and after treating overlaps calculates statistics and generates the output file.

### 2.3. Algorithms

The ProGeRF algorithm receives as input parameters (a) a (multi)FASTA file, (b) minimum size of the repetitive pattern, (c) the minimum and maximum sizes of the motif (word DNA or amino acids length or sliding window length), (d) maximum amount of gaps accepted between each motif of a repeat, (e) percentage of maximum degeneration accepted for a motif, (f) overlapping percentage, and (g) run mode, that is, whether using a FASTA file of DNA or of amino acids.

RepeatFinder procedure executes, in parallel, for each motif size within the range of minimum and maximum values, to identify sequences with all motif lengths in this range of values in the FASTA file.

**Figure 2:**
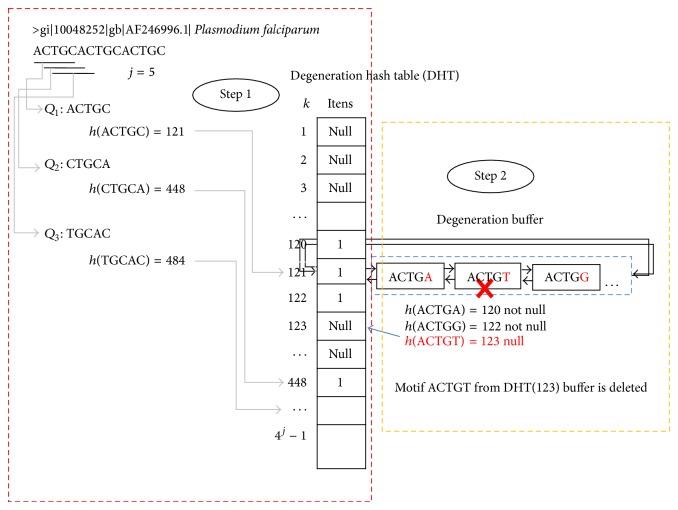
Creating degeneration hash table: Step  1: sliding window maps each motif of the sequence for a position in the degeneration hash table and sets value 1 to mapped position. Step  2: generate possible degeneration of the sliding window and store in the buffer at position *k* of the sliding window; only the degeneration that mapped to a position of the hash table presents a value of 1.

An overview of the ProGeRF algorithm is as shown in Figure 2.

**Figure 3:**
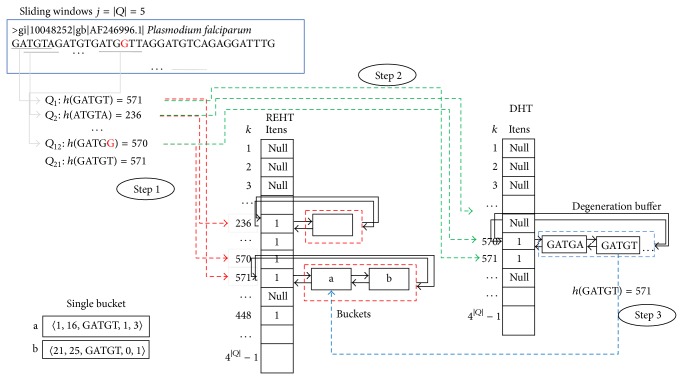
Creating repetitive element hash table: Step  1: sliding window maps each motif of the sequence for a position in the repetitive element hash table and sets value 1 to mapped position, and add or remove the sliding window to single bucket; Step  2: check whether the current sliding window is a degeneration of some motif ever recorded in buckets of REHT; Step  3: for each existing degeneration in the buffer the function *h*() is applied and then converted into an integer *k* and, soon after, Step  1 is performed.

1. Dynamically allocate two hash tables (repetitive element hash table and degeneration hash table) of radix^|*Q*|^ positions, where radix is four for DNA and twenty for amino acids and |*Q*| motif length. Each position in the tables is mapped to a unique combination of nucleotide/amino acids of length |*Q*|.
2. Read the first sequence from FASTA file.
3. Creating degeneration hash table* (DHT)*: for each sliding window *Q*_*p*_, along the first sequence, where *p* = 1,2, 3,…, *n* − *j* + 1, *n* is the sequence length and *j* is the sliding window size (*j* = |*Q*|). RepeatFinder procedure converts each *Q*_*p*_ to an integer key *k*, as previously discussed. With this, the position *k* of DHT is set to 1; this process is illustrated by Figure 2, Step  1.
4. Repeat the previous process for *p* = 1,2, 3,…, *n* − *j* + 1, where *n* is the sequence length and *j* is the sliding window size.
5. For each position marked with 1 in the degeneration hash table, run the generating degeneration procedure. This procedure generates all possible degenerations of a motif up to a maximum percentage of defined imperfection. Each motif degenerate generated is converted to an integer key *k*′, and if at position *k* of the degeneration hash table is marked as 1 the motif degenerate generated is inserted into a degeneration buffer. Otherwise, if position *k*′ is marked as null, the motif is not inserted in the degeneration buffer, Figure 2, Step  2.
6. Creating repetitive elements hash table (REHT), illustrated by Figure 3: for each sliding window *Q*_*p*_, where *p* = 1,2, 3,…, *n* − *j* + 1, do the following:
  - calculate *k* = *h*(*Q*_*p*_);
  - Step  1: if a single bucket does not exist at position *k* of REHT then create a single bucket and set *sp* and *fp* with the initial and final positions of the motif *Q*_*p*_. However, if there is a bucket and 0 < *p* − *fp* ≤ *ga* (where *p* is sliding window position, *fp* the value registered in the bucket final position, and *ga* the maximum gap allowed between motif), then
    - set *fp* = *p* + |*Q* | −1, that is, the final position of the current sliding window;
    - increase the field *lt* of bucket;
    - set *gp* = *gp* + *p* − *fp*; that is, record the total number of gaps.
  - However, if there is a single bucket and the condition 0 < *p* − *fp* ≤ *ga* is not satisfied and if the bucket field *lt* is not greater than or equal to the minimum amount of repetitions defined, then the last single bucket is deleted.
  - Step  2: check whether the current sliding window is a degeneration of some motif ever recorded in buckets of REHT. For this, degeneration buffer at position *k* of degeneration hash table is traversed.
  - Step  3: for each existing degeneration in the buffer, the function *h*() is applied and then converted into an integer *k*, after which Step  1 is performed. However, the single bucket is not deleted if the condition 0 < *p* − *fp* ≤ *ga* is not satisfied.
7. Save the REHT results in a file and later erase its data.
8. Repeat steps  1–7 for the other sequences in the FASTA file.
9. In dealing with overlaps, join all the files from step  7 into a single file, sort the rows by the initial position of the repetition and for each row that represents a repetitive element, and check the following:
  - if the current repetitive element has an initial position less than the final position of the previous repetitive element then compute the degree of overlap;
  - if the degree of overlap is within the permitted value, skip to the next repetition. Otherwise, delete the smallest repetitive element and pass on to the next line.
10. Print the remaining reps in the file.

### 2.4. Implementation

Hash tables were developed to perform a dynamic allocation of memory which allows the program to read FASTA files of any size. Furthermore, degeneration buffer and the buckets were implemented through circular doubly linked lists, which allow you to insert or remove degenerations or single buckets in the hash table quickly, without the need to scroll through the whole list.

Time complexity to create the degeneration hash table is approximately linear in function of the number of nucleotides or amino acids sequence, because the algorithm runs once the input sequence to identify the existing motif, scoring with 1 the position in the degeneration hash table of motif found. Then, it traverses the degeneration hash table, and at positions marked with 1, the possible degenerations are generated for the corresponding motif.

The algorithm accepts a maximum of 35% degeneration, that is, at most two degenerate characters in a motif of size 7. The amount of possible combinations for a motif of size *j* with degeneration by up to 2 characters is given by

(2) $$\begin{array}{c} c=\frac{1}{2}\left({\left(r-1\right)}^{2}j^{2}+\left(r-1\right)j\right), \end{array}$$

where *r* is the radix (four for DNA and twenty for amino acids) and *j* = |*Q*|, that is, the length of the word (in our case it is the sliding window length). Because *c* does not vary with the size of the input sequence, it can be considered constant, so the time complexity to generate the degeneration hash table is of the order *O*(*n*).

The step of generating REHT also presents linear time complexity depending on the size of the sequence input. Because the sliding window traverses the FASTA sequence once for every sliding window, the corresponding motif is inserted or deleted in the bucket in constant time and then tested at most *c* possible degenerations, and as *c* can be considered constant, we have time complexity in order *O*(*n*). Therefore, the RepeatFinder procedure presents time complexity of the order *O*(*n*).

### 2.5. Interface and Output

ProGeRF is designed to have web and command line interface. The command line interaction may be performed by indicating the (multi)FASTA file address containing DNA or amino acids sequence(s), the motif length range, the minimum repeated times for all motif lengths or the minimum repeated time for each one, the maximum gaps allowed between motifs, the maximum degeneration percentage, the motif shifting percentage, and the run mode that defines DNA or amino acids input sequence and the output file name.

For example, the command sequence perl progerf.pl −q Linfantum_JPCM 5.FASTA −o output −i 2 −y 6 −r 5 −g 3 −v 0 −d 20 −m n will search repetitive elements in the file Linfantum_JPCM5.FASTA of motif with length range between 2 and 6, with maximum gaps of 3, motif overlap of 0%, degeneration of 20%, and run mode of nucleotide, and the result will be saved in the output file.

The results file presents a table, wherein each column represents the following information in order: sequence ID, size of the DNA/protein sequence, minimal repetitions allowed, repetition amount, repetitive element start and final position, number of gaps, statistics (only nucleotide run mode), and complete repetitive element.

The web mode, available at http://64.79.105.19/ligp/, offers a user-friendly interface developed using bootstrap packages for layout formatting, a JBrowse plugin [22] and jqGrid [23]. Web interface provides the same flexibility as command line mode. However, it is platform independent and can be run in any browser; the parameter setting is performed through forms, buttons, text boxes, and a combo box.

Web interface provides three ways of sending the FASTA file containing DNA or amino acid sequences.

1. File upload: the users can send a FASTA file from their own computer.
2. Copy and paste sequence: the user copies a sequence of interest and pastes in the text box.
3. Automatic download from the NCBI data base: the user enters one or more GI numbers separated by commas, and the tools will download the sequences from the NCBI data base and run the repetition extraction algorithm. GI number (GenInfo identifier) is a unique number that identifies a particular sequence in the NCBI databases.

The results on the web page can be viewed in two ways: tabular format using the jqGrid script and graphical format, through the JBrowse plugin [22].

jqGrid is an Ajax-enabled JavaScript control that provides solutions for representing and manipulating tabular data on the web dynamically [23]. With jqGrid, the user can make queries for a particular motif pattern, setting several query filters and sorting the results by any of the columns.

JBrowse is a browser for genome viewing, developed in JavaScript, in which the user can navigate through the genome annotations on the web. In JBrowse, it is possible to zoom, navigate, and select range of subsequence within a genome [22].

## 3. Results and Discussion

We present two experiments in this paper. The first experiment demonstrates the efficiency of ProGeRF compared with other microsatellite identification tools, and the second experiment demonstrates the use of the repetitive element identification algorithm in protein FASTAs files.

Our current implementation features a Dell Inspiron, Intel core 2 duo 2.2 GHz processor with 2 MB cache, 3 GB RAM, 320 GB hard drive, and the Ubuntu operating system 14.04 LTS 32 bits.

For the first experiment, the tools Misa [13], Mreps [14], GMATo [19], SciRoKo [15], Sputnik [16], TRF [11], and ProGeRF were executed on each of the following genomic sequences:* Plasmodium falciparum* chromosome IV (NC_004318.1),* Saccharomyces cerevisiae* chromosome IV (NC_001136.8),* Mycobacterium tuberculosis* H37Rv genome (NC_000962.2) used in the work of Mudunuri and Nagarajaram [18] downloaded from ftp://ftp.ncbi.nih.gov/genomes, and the whole* Setaria italica* genome used in the work of Wang et al. [19], download from phytozome http://www.phytozome.net/.

For tools that allow for configuring the parameters minimum size, maximum size, and a minimum number of repetitions of five motifs, the values set for these parameters were 1, 6 and 5, respectively. For the remaining parameters, the following values were used according each tool: (a) Misa: maximum difference between 2 SSRs of 0; (b) Mreps: a resolution of 5; (c) SciRoKo: mode mismatched fixed penalty, with other parameters' score using default values; (d) Sputnik: a maximum size of 5 (maximum allowed by the tool), a minimal score: 5, maximal recursion: 0, minimum length of SSR to report: 10, and points for a mismatch and points for a match: 1; (e) TRF: matching weight: 2, mismatching penalty: 7, indel penalty: 7, match probability: 80, indel probability: 10, Minscore: 2, and MaxPeriod: 15; and (f) ProGeRF: a maximum number of gaps allowed 1, overlap of 0%, a degeneration of 20, and nucleotide mode.

IMEX tool presented error during the execution of the versions 1.0 and 2.0 in Ubuntu operating system 14.04 of 32 bits; thus it has not been possible to compare the results of this tool. In the first three sequences, Table 1, ProGeRF was a little slower than SciRoKo, Sputnik, Misa, and Mreps. However, the time can still be considered good, if we note the much larger number of repetitions tracked than the other tools. The number of repetitive elements of tools SciRoKo, Sputnik, and Mreps are smaller than of tools Misa, GMATo, TRF, and ProGeRF, but GMATo is slower than Misa, TRF, and ProGeRF. It is important to mention that GMATo tool is nonspecific in its treatment of overlaps and Wang et al. [19] relate that the extra loci from Misa are mined redundantly in the overlapped microsatellites.

The smaller numbers of repetitive elements found by tools SciRoKo, Sputnik, and Mreps are due to the fact that (a) Sputnik does not report hexanucleotide since maximum allowed is pentanucleotide; (b) according Mudunuri et al. [8] score based tools as SciRoKo and Sputnik that use higher mismatch penalties (such as 5, 6, and 7) and less match weights (such as 1, 2) fail to identify many smaller microsatellites (mono-tri); and (c) Mreps is highly constrained by its internal minimum size threshold, since detection starts at 11 bp for dinucleotides, 12 bp for trinucleotides, and up to 15 bp for hexanucleotides [14, 15, 18].

For three files, Table 1, a smaller number of repetitive elements has been identified by ProGeRF compared with the TRF tool, approximately 118 thousand differences in number. However, the TRF tool allows the occurrence of overlap where the redundancy is, at most, three pattern sizes and therefore presents a much larger number of repetitions than ProGeRF.

By default, ProGeRF does not allow overlaps and chooses the biggest repetitive elements sequence. However, the user can define the overlap percentage allowed, through the parameter −v. Nevertheless, the runtime of ProGeRF was lower than TRF and 7 times smaller than that of GMATo.

We evaluated whether the detections returned by tools on sequences NC_004318.1, NC_001136.8, and NC_000962.2, Table 1, occur at the same physical locations in genomes. More than 75% of SciRoKo, Sputnik, and Mreps detections are also detected by ProGeRF on the three sequences and GMATo and Misa detections are full coverage by ProGeRF on the three sequences, Table 2.

Sputnik and TRF present low amount of loci covered by ProGeRF on sequences NC_001136.8 and NC_000962.2. This low coverage is consequence of the lack of a parameter to set maximum size and minimum number of repetitions, which allows them to find a larger number of repetitive elements.

Therefore, we filter the results of Sputnik and TRF tools, limiting the results to minimal repeat of 5, minimal size of 1, and maximum size of 6. Thus, ProGeRF coverage increases to 100% over results of Sputnik and more than 80% over results of TRF (97% for sequences NC_004318.1 and NC_001136.8).

On the other hand, the coverage of ProGeRF by SciRoKo, Mreps, and Sputnik is lower than 46% for all sequences and much lower than 9% when observing the last two sequences. This is consistent with the fact that ProGeRF detects more repetitive elements than others tools.

In the second experiment, we run the ProGeRF web version in the protein mode in circumsporozoite protein (ACO49545.1), merozoite surface protein 1 (XP_001352170.1), and merozoite surface protein 9 (AAN36363.1).

**Figure 4:**
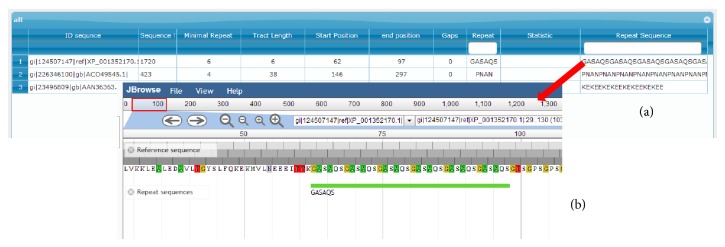
Screen shot from circumsporozoite protein (ACO49545.1), merozoite surface protein 1 (XP_001352170.1), and merozoite surface protein 9 (AAN36363.1) element repetitive search: (a) visualization of results through the jqGrid plugin: by clicking over the repetitive element the graphical view is opened; (b) repetitive elements are mapped and displayed graphically through JBrowse.

Table 3 presents the result of executing the circumsporozoite protein (ACO49545.1), merozoite surface protein 1 (XP_001352170.1), and merozoite surface protein 9 (AAN36363.1), in which the repetitive element PNAN (PRO-ASN-ALA-ASN) was identified in the circumsporozoite protein as in previous work [24]. In other proteins, repetitive elements have been identified with low repetition frequency.  Figure 4 shows the result that is available to the user in the web environment: (A) visualization of results through the jqGrid plugin: clicking over the repetitive element opens the graphical view; (B) repetitive elements are mapped and displayed graphically through JBrowse. In the web environment an identification code is generated for each execution. The code can be used to review the result when necessary and it is still possible to receive a link with the code by email to notify the user.

Regarding the tool in web mode, no other web tool offers the user the possibility to consult executions previously carried out and the integration/visualization of results using a dynamic and friendly environment for navigation genome with jBrowse.

## 4. Conclusion

ProGeRF, the proposed identification algorithm for repetitive elements, presents itself as an efficient, fast, accurate, and easy to use tool and is available in either stand-alone or web mode. It offers a dynamic and user-friendly web interface, the identification of perfect and imperfect repetitive elements, repeat size detection from 1 to 12, repeating the search for specific sizes, preview of the alignment, the flanking sequence, repetition statistics, and a graphical output.

Among the tools that locate both perfect and imperfect repeats ProGeRF is the one that provides graphical visualization and allows for the filtering of the results. Another advantage is the possibility of executing it on genomic and proteomic data and the ability to treat large genomic/proteomic data files.

## Figures and Tables

**Table 1:** Comparison of amount detection and execution times (in seconds) of Mreps, Misa, Sputnik, GMATo, SciRoKo, TRF, and ProGeRF. The features were run on a Dell Inspiron, Intel core 2 duo 2.2 GHz processor with 2 MB cache, 3 GB RAM, 320 GB hard drive, Ubuntu operating system 14.04 LTS 32 bits.

Sequence	Mreps	Misa	Sputnik	GMATo	SciRoKo	TRF	ProGeRF
	Rep (time)	Rep (time)	Rep (time)	Rep (time)	Rep (time)	Rep (time)	Rep (time)
NC_004318.1 (1204 kb)	9608 (2.8)	22867 (3.2)	7420 (0.7)	23539 (10.3)	3763 (1.1)	30244 (72.4)	26164 (3.9)
NC_001136.8 (1531 kb)	935 (1.4)	10640 (3.3)	1427 (0.9)	10721 (7.7)	185 (0.7)	8101 (4.5)	11552 (2.4)
NC_000962.2 (4411 kb)	1412 (3.9)	6832 (8.9)	3140 (1.46)	6846 (12.1)	72 (1.5)	19496 (24.5)	11422 (4.0)
*Setaria* ^*^ ($\tilde{5}$15 Mb)	— (—)	2054241 (868.3)	480644 (105.7)	2073643 (9859.1)	47770 (129.0)	2438036 (1481.5)	2319812 (1352.0)

^*^Whole genome. The value in brackets is the runtimes in seconds.

**Table 2:** Loci and nucleotide coverage between tools.

Sequence		B							
		Tools	Mreps	Misa	Sputnik	GMATo	SciRoKo	TRF	ProGeRF
*Plasmodium* Chr4 NC_004318.1	A	Mreps	—	78 (45)	53 (33)	78 (45)	41 (36)	98 (74)	89 (60)
		Misa	47 (63)	—	21 (4)	100 (99)	18 (40)	88 (79)	100 (98)
		Sputnik	91 (86)	70 (74)	—	70 (74)	58 (69)	0 (0)	83 (84)
		GMATo	49 (62)	100 (99)	21 (40)	—	19 (40)	89 (79)	100 (98)
		SciRoKo	96 (95)	93 (76)	95 (71)	92 (76)	—	95 (96)	98 (91)
		TRF	51 (41)	54 (32)	0 (0)	54 (32)	16 (21)	—	68 (46)
		ProGeRF	46 (56)	86 (67)	21 (31)	86 (67)	16 (32)	87 (78)	—

SAC Chr4 NC_001136.8	A	Mreps	—	60 (40)	42 (26)	68 (40)	18 (20)	95 (74)	86 (62)
		Misa	7 (12)	—	3 (7)	100 (99)	1 (4)	33 (37)	100 (99)
		Sputnik	30 (35)	29 (30)	—	29 (30)	12 (1)	74 (73)	38 (39)
		GMATo	7 (12)	100 (99)	3 (7)	—	1 (4)	33 (37)	100 (99)
		SciRoKo	91 (89)	77 (61)	86 (59)	77 (61)	—	99 (99)	94 (72)
		TRF	15 (16)	41 (26)	13 (12)	41 (26)	2 (5)	—	48 (35)
		ProGeRF	8 (16)	92 (80)	4 (7)	92 (80)	1 (5)	34 (39)	—

MTB H37Rv NC_000962.2	A	Mreps	—	9 (3)	15 (7)	9 (3)	3 (3)	91 (71)	75 (58)
		Misa	2 (3)	—	1 (1)	100 (99)	0.5 (1)	13 (13)	100 (99)
		Sputnik	6 (7)	2 (2)	—	2 (2)	1 (1)	66 (64)	14 (14)
		GMATo	2 (3)	100 (99)	1 (1)	—	0.5 (1)	13 (13)	100 (99)
		SciRoKo	73 (74)	47 (36)	63 (41)	47 (36)	—	100 (100)	79 (72)
		TRF	8 (7)	4 (1)	10 (7)	4 (1)	0.4 (0.5)	—	18 (13)
		ProGeRF	9 (16)	60 (35)	4 (4)	60 (35)	0.5 (1)	29 (33)	—

Percentage of the total number of detections (perfect and imperfect) of tools A also detected (i.e., covered) by tools B. The value in brackets is the proportion of nucleotides detected by A and covered by B.

**Table 3:** Repetitive protein elements found by the web tool ProGeRF.

ID sequence	Locus	Motif	Rep.

*XP_001352170.1 *	62–97	GASAQS	6
*ACO49545.1 *	146–297	PNAN	38
*AAN36363.1 *	693–712	KEKEE	4

The parameters used were motif size between 2 and 6, repetitions of the least 4 motifs, and zero for the gaps, overlap, and degeneration.
